# Complete Genome Sequences of *Arthrobacter* Phages Beans, Franzy, Jordan, Piccoletto, Shade, and Timinator

**DOI:** 10.1128/genomeA.01094-17

**Published:** 2017-11-02

**Authors:** Tamarah L. Adair, J. Alfred Bonilla, Karen K. Klyczek, Julia Y. Lee-Soety, German Rosas-Acosta, Melinda Harrison, Baylor University SEA-PHAGES, University of Wisconsin–River Falls SEA-PHAGES, Saint Joseph’s University Phage Safari, The University of Texas at El Paso SEA-PHAGES, Cabrini University SEA-PHAGES, Charles A. Bowman, Steven G. Cresawn, Rebecca A. Garlena, Daniel A. Russell, Welkin H. Pope, Deborah Jacobs-Sera, Graham F. Hatfull

**Affiliations:** a Department of Biology, Baylor University, Waco, Texas, USA; b Biology Department, University of Wisconsin–River Falls, River Falls, Wisconsin, USA; c Department of Biology, Saint Joseph’s University, Philadelphia, Pennsylvania, USA; d Department of Biological Sciences, the University of Texas at El Paso, El Paso, Texas, USA; e Department of Science, Cabrini University, Radnor, Pennsylvania, USA; f The Scripps Institute, La Jolla, California, USA; g Biology Department, James Madison University, Harrisonburg, Virginia, USA; h Department of Biological Sciences, University of Pittsburgh, Pittsburgh, Pennsylvania, USA

## Abstract

We report here the genome sequences of six newly isolated bacteriophages infecting *Arthrobacter* sp. ATCC 21022. All six have myoviral morphologies and have double-stranded DNA genomes with circularly permuted ends. The six phages are closely related with average nucleotide identities of 73.4 to 93.0% across genomes lengths of 49,797 to 51,347 bp.

## GENOME ANNOUNCEMENT

Here, we report complete genome sequences of six bacteriophages infecting *Arthrobacter* sp. ATCC 21022 (1), isolated as part of the Science Education Alliance-Phage Hunters Advancing Genomics and Evolutionary Science (SEA-PHAGES) program (2). Many *Arthrobacter* spp. are soil bacteria and can break down complex hydrocarbons, which can be useful in bioremediation (3–5). Phages Beans, Franzy, Jordan, Piccoletto, Shade, and Timinator were obtained from soil samples collected from River Falls, WI; Pittsburgh, PA; Radnor, PA; Cloudcroft, NM; King of Prussia, PA; and Frisco, TX, respectively. All were isolated by enrichment cultures and appear to be lytic phages, forming small, clear plaques. By electron microscopy analysis, all belong to the *Myoviridae* family, with contractile tails approximately 100 nm long and isometric heads approximately 50 nm in diameter.

The genomes were sequenced using the Illumina shotgun sequencing method at either the University of Pittsburgh or at North Carolina State University Genomic Sciences Laboratory. The sequences were assembled using Newbler, generating single major contigs with coverage from 117-fold to 3,520-fold. The observed genome sizes range from 49,797 bp (Beans) to 51,347 (Jordan), with G+C contents ranging from 60.9% (Timinator) to 63.6% (Beans and Piccoletto). All of the phages have circularly permuted ends, and coordinate position one was assigned to the first nucleotide of the predicted gene immediately upstream of the terminase large subunit gene, which is a strong candidate for encoding a terminase small subunit containing a helix-turn-helix DNA binding domain.

Genomes were annotated using DNA Master (http://cobamide2.bio.pitt.edu), and coding sequences were predicted using GeneMark (6) and Glimmer (7); no tRNA genes were identified using Aragorn (8) and tRNAscan-SE (9). Functional assignments were made using BLASTp (10) and HHpred (11, 12) against the publicly available GenBank, Protein Data Bank, and Pfam databases. The genomes share significant nucleotide similarity, with average nucleotide identities of 73.4 to 93.0%, and all six phages are grouped into Cluster AO, along with the previously described phages BarretLemon, Brent, Jawnski, Martha, Sonny, and TaeYoung (13).

The genomes contain 73 to 79 protein-coding genes, of which approximately 30% have predicted functions. The virion structural genes are organized canonically, and include tail sheath and baseplate proteins as expected for myoviral phages. The genomes contain a single endolysin-coding gene with peptidase-, amidase-, and peptidoglycan-binding domains similar to mycobacteriophage lysin A proteins (14), but do not have lysin B genes. As predicted, no integrase, immunity repressors, or other genes associated with lysogeny were identified. All of the genomes code for recombination systems, including an exonuclease and a RecT-like recombinase, as well as RusA-like Holliday junction resolvases, that could be involved in concatemerization and genome circularization (15–18). Genes encoding AlpA-like DNA binding proteins, and DNA polymerase III beta subunits are also present.

### Accession number(s).

Complete genome sequences are available in GenBank under the accession numbers MF324907 (Beans), MF377442 (Franzy), MF189176 (Jordan), MF189177 (Piccoletto), MF189178 (Shade), and MF377441 (Timinator).
